# OMICs in Cariology and Endodontology – what have we learned so far?

**DOI:** 10.1080/20002297.2017.1325192

**Published:** 2017-05-25

**Authors:** Egija Zaura

**Affiliations:** ^a^ University Research Chair Professor in Oral Microbial Ecology at Vrije Universiteit Amsterdam, Amsterdam, the Netherlands;; ^b^ ACTA

## Abstract

During the last decade, OMICs techniques have conquered the field of oral microbiology. This has resulted in an exponential growth of publications on microbiome related to different stages of caries process, as well as on primary and recurrent root canal infections. But have these technological advances brought us any groundbreaking insights that would help a clinician in choosing the right treatment or prevention strategies? In this presentation the current knowledge on microbiome related to Cariology and Endodontology fields will be critically assessed. Then the potential pitfalls and shortcomings of the studies using OMICs approaches will be discussed and future directions will be highlighted.

